# Actively ventilating calf hutches using solar-powered fans: Effects on hutch microclimate and calf thermoregulation

**DOI:** 10.3168/jdsc.2023-0390

**Published:** 2023-10-06

**Authors:** Bethany Dado-Senn, Jennifer Van Os, Joao Dorea, Jimena Laporta

**Affiliations:** Department of Animal and Dairy Sciences, University of Wisconsin–Madison, Madison, WI, 53597

## Abstract

Although active ventilation via fans is an effective and widely adopted heat abatement method for use with adult dairy cattle, it has yet to be investigated in outdoor hutch-housed dairy calves despite most US calves being raised in such systems. We investigated a solar-powered fan system for outdoor calf hutches and its effect on hutch microclimate and calf thermoregulation. During summer, a 3 × 3 Latin square was replicated 4 times (n = 12 preweaning heifers) with 4-d exposure periods to minimally (CON; rear windows closed), passively (PASS; rear windows opened), or actively (ACT; solar-powered fan, activated at dry bulb temperature [T_db_] > 21°C) ventilated hutch systems. Hutch microclimate and calf thermoregulation were evaluated either continuously (T_db_, humidity, rectum surface temperature, and behavior) or after a daily 30-min inside restriction (air speed, air particle number, noise level, respiration, and sweating rate, and skin and rectal temperature). Active ventilation substantially increased hutch air speed relative to PASS and CON (1.76 vs. 0.19 vs. 0.05 m/s). However, PASS hutches had the lowest INT T_db_ (27.2 vs. 26.4 vs. 27.8°C), whereas ACT INT T_db_ was reduced at 0900 and 1000 h relative to CON but not PASS. Similarly, ACT reduced calf respiration rates and lowered rectum surface temperature at 0800 and 0900 h when compared with CON but not PASS. The lack of strong ACT influence on calf outcomes over PASS could partially be explained by the decreased proportion of time ACT calves spent inside their hutch (48.7 vs. 67.3 vs. 64.1% of each hour). Overall, ACT improved hutch microclimate and calf responses relative to CON but not PASS. Either ACT or PASS ventilation may be sufficient to provide heat abatement to continental hutch-housed calves.

## Highlights

• Environmental temperature averages remained above 21°C for 84% of the day.
• Calf hutch active ventilation via solar-powered fans led to air speeds of 1.7 m/second.
• Active and passive ventilation reduced hutch internal temperature.
• Actively ventilated calves had reduced respiration versus minimally ventilated calves.
• Actively ventilated calves spent less time in the hutch and more time lying outside.

## Graphical Abstract

**Figure:**
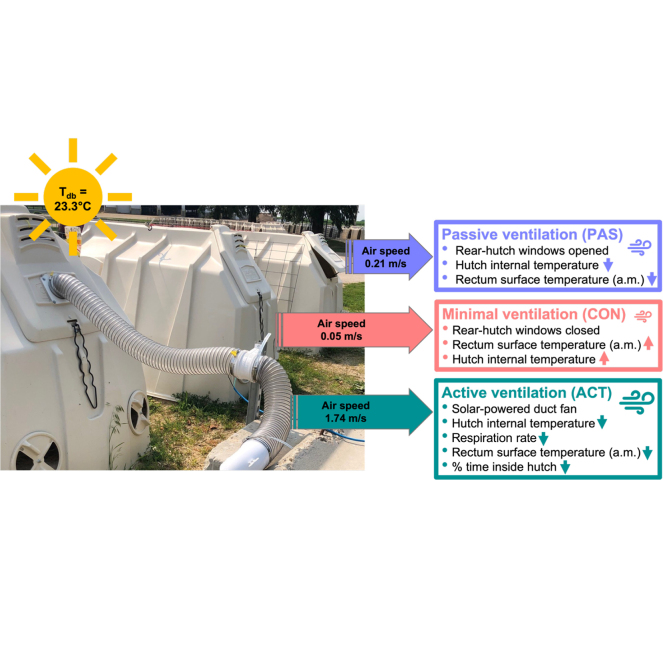
**Summary:** Three ventilation strategies were investigated for outdoor, hutch-housed dairy calves during a continental summer with varying effects on hutch microclimate and calf thermoregulatory responses.

Despite greater thermotolerance and reduced heat loads relative to mature dairy cattle, preweaning dairy calves are susceptible to productive, health, and welfare impairment under heat stress (Broucek et al., 2009; Wang et al., 2020a). Calf heat stress occurs at an upper critical temperature between 26°C and 32°C, depending on the calf's environment, age, size, and metabolic status (Neuwirth et al., 1979; Hahn, 1981). A more recent analysis of calf animal-based responses under rising ambient temperatures indicates that calves raised in a continental climate begin to show signs of thermal discomfort at a lower threshold of 21°C (Dado-Senn et al., 2023). Because many regions reach these benchmarks in the summer and the global temperature continues to rise, there is an emergent need for sustainable heat abatement solutions for dairy calves (Van laer et al., 2014; Beck et al., 2018).

Active heat abatement via mechanical ventilation has been proven effective in mitigating heat stress in mature dairy cattle. Cow-side target air speeds between 1.0 to 2.4 m/s are suggested to promote cattle thermoregulation, lying behaviors, and milk yields (Reuscher et al., 2021a). Conversely, dairy calves are primarily provided passive ventilation, as the optimal mechanisms for active heat abatement have yet to be elucidated. Limited studies have tested passive heat abatement via outdoor hutch modification or supplemental shade (Coleman et al., 1996; Moore et al., 2012; Bakony et al., 2021), and few studies have evaluated indoor forced ventilation. Providing fans to indoor, individually housed calves in a temperate climate promoted ADG and reduced respiration rates (Hill et al., 2011). Similarly, subtropical group-housed calves with fan access at air speeds of 2 m/s had improved thermoregulation, DMI, and health relative to calves provided shade and natural ventilation at air speeds of 0.15 m/s (Dado-Senn et al., 2020).

Although most dairy calves in the United States are housed in hutch systems (~63%; 25% indoor and 38% outdoor; NAHMS, 2016), active heat abatement through fans has yet to be investigated for this system. This is partly due to the feasibility of powering fans to individual calf hutches and the lack of research on the design and necessity of active heat abatement for dairy calves. To address these challenges, we performed a pilot study investigating a solar-powered fan system in individual outdoor calf hutches to provide active ventilation for heat abatement. The study objectives were to evaluate minimally, passively, and actively ventilated calf-hutch systems on hutch microclimate and animal-based responses. We hypothesized that the increased air speed inside the hutch resulting from active relative to passive or minimal ventilation would improve air quality and reduce internal hutch temperature with consequent improvement of calf thermo-physiological and behavioral responses.

All procedures were approved by the University of Wisconsin–Madison Institutional Animal Care and Use Committee (study #A006455). This study was conducted from July 12 to 30, 2021, at the University of Wisconsin–Madison Arlington Research Station (Arlington, WI). The experiment was a 3 × 3 Latin square replicated 4 times (n = 12 female Holstein dairy calves) with a 4-d treatment exposure period and a 3-d resting period in between. The rationale for the 4-d treatment duration was based on previous studies of calf heat stress that studied calf thermoregulatory responses (Kovács et al., 2020). The resting period was employed to “wash out” any residual effect of ventilation treatment on calf or microclimate outcomes before starting the next treatment period. Calves were randomly assigned treatment within replicates at the start of the experiment. On Monday of each week, treatments were initiated at 1600 h for initial exposure. Data were collected from Tuesday to Friday, and treatments were halted at 1400 h on Friday with the resting period from Friday night until Monday morning.

Calves born sequentially in mid-June 2021 were selected for this study. Calves were weighed at birth (38.5 ± 4.8 kg), fed 3.78 L of colostrum, and thereafter fed 7.6 L/d milk replacer (Cow's Match, Land O'Lakes Animal Milk Products Co.) at 0400 and 1500 h. Starter concentrate and water were offered ad libitum inside and outside the hutch, respectively. Calves were enrolled at 22 ± 5 d of age (mean ± SD) for a total of 21 d to avoid early-life scours and weaning, which started at 49 d of age.

Calves were individually housed outdoors in sand-bedded polyethylene calf hutches (Calf-Tel, L. T. Hampel Corp., Germantown, WI; 2.1 × 1.2 × 1.4 m; length × width × height) with the option for rear-hutch ventilation through an upper panel and 2 lower window vents (diameter ~30.5 cm; Figure 1A). All calves had free access to a wire-enclosed pen (1.7 × 1.4 m) except at the time of restriction (described below). Calves remained in their individual hutch for the duration of the study, and different treatments were applied to the hutch in each period. Treatments were considered either minimal ventilation (**CON**; all rear-hutch ventilation closed), passive ventilation (**PASS**; rear-hutch upper panel opened to a 20° angle and lower window vents opened), or active ventilation (**ACT**; solar-powered fan placed in closed upper panel and lower window vents opened). Each replicate was grouped to make a line of 12 calf hutches, behind which 4 solar panels were placed (Figure 1C). Treatment designs are visualized in Figure 1A–C.

**Figure 1:**
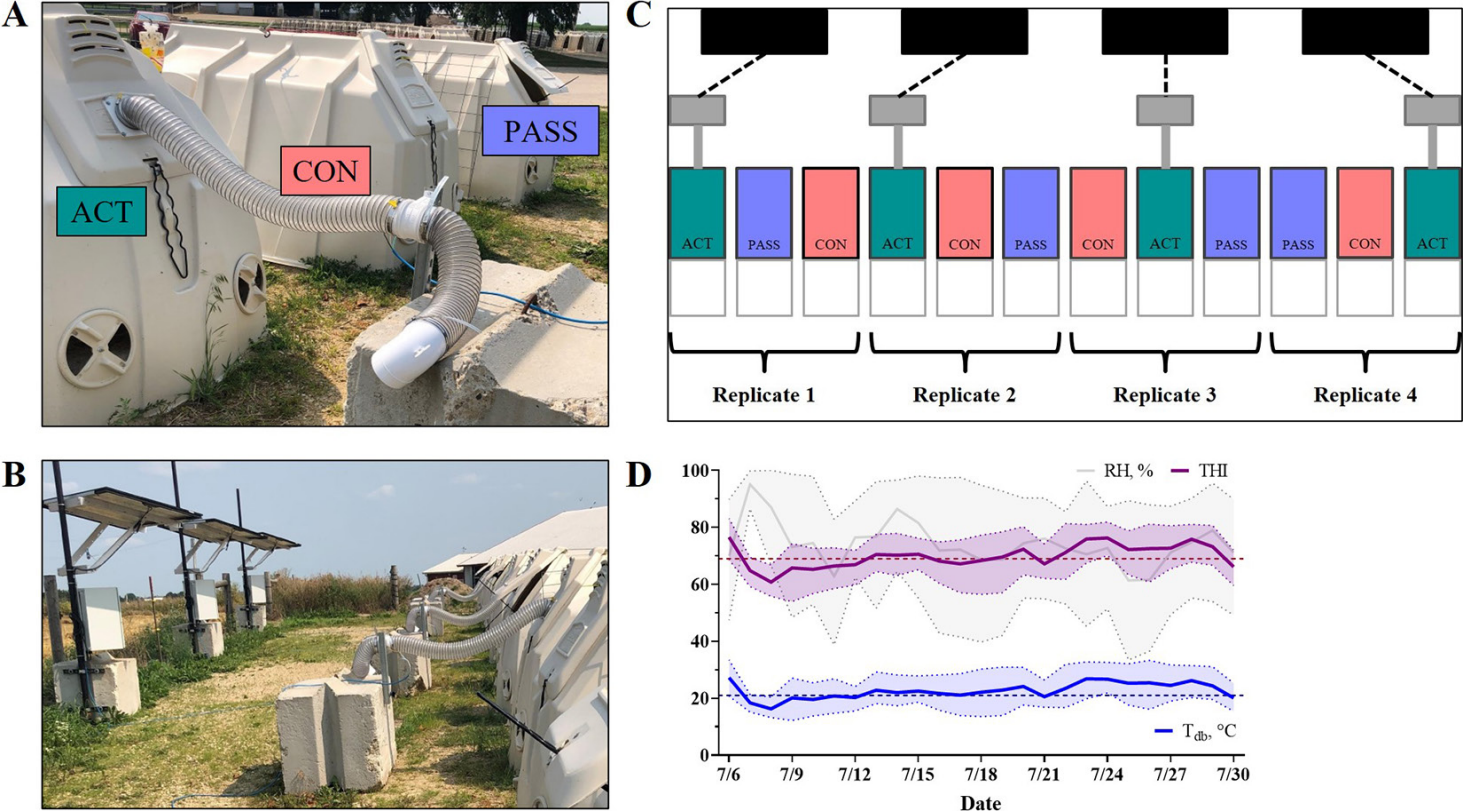
Outdoor calf hutch ventilation treatment designs. (A) Pictographic representation of active (ACT, solar-powered fans activated at 21°C), minimally (CON, rear windows closed), and passively (PASS, rear windows opened) ventilated calf hutch treatments. (B) Solar-power design for ACT hutches. (C) Layout of 12 hutches, fans, and solar panels. Hutches depict treatment randomization within replicates. (D) External environmental parameters across the duration of the study: dry bulb temperature (T_db_), relative humidity (RH %), and temperature-humidity index (THI). Shading indicates the SD of the mean.

The active ventilation system was designed and installed in coordination with a solar-panel engineering company (Ag Video Surveillance, Beaver Dam, WI) using weatherproof outdoor solar panels (model #RPST12/24M-200–255LE; Tycon Solar) with 165 × 102 cm dimensions, 50 W output, and operating temperature between −30°C and +60°C. Solar panels were wired to duct fans (12 V, Attwood 1749–4 Turbo 4000 Series) with a 0.2-m outlet tube (10.2 cm diameter) and a 0.6-m tube running from the fan to the back of the hutch. This tubing was secured to a hole in the rear upper panel of the calf hutch (Figure 1A). Fans were controlled by a thermostat located externally at the base of the shaded panel electronics system that activated fans at a temperature of 21°C.

Hutch external (**EXT**) environmental measures, including dry bulb temperature (**T_db_**) and relative humidity (**RH**), were recorded every 15 min across the duration of the study and averaged hourly using HOBO Pro Series Temp Probes (Onset Computer Corp.) attached to an external structure near the hutch-housing. From these values, the temperature-humidity index (**THI**) was calculated (NRC, 1971) and averaged hourly. Hutch internal (**INT**) microclimate environmental measures of T_db_, RH, and THI were also calculated every 15 min and averaged hourly using Kestrel DROP D2AG monitors (Kestrel Instruments) affixed to the top-rear of each calf hutch. This study will primarily report T_db_ environmental outcomes instead of THI, as ambient temperature may be a better indicator of heat stress for dairy calves raised in a continental climate (Dado-Senn et al., 2023). Temperature probes (i-button DS1922-F5#; Maxim) were attached along the calf rectum to assess calf rectum surface temperature every 15 min and averaged hourly for analysis, according to Dado-Senn et al. (2020).

During treatment d 1 to 4, calves underwent in a restriction period to standardize calf environments and determine the effects of ventilation strategy on calf physiological responses and hutch microclimate. Starting at 1200 h, calves were subjected to a 30-min EXT restriction followed by a 30-min INT restriction using wire paneling and bungee cords to keep calves out or in during restrictions. Calf respiration and sweating rates (**RR**, **SR**) and rectal and skin temperatures (**RT**, **ST**) were measured at the end of each 30-min restriction and the ΔINT-EXT was calculated. Animal thermoregulatory responses were assessed according to Davidson et al. (2022). While calves were restricted EXT, hutch microclimate measures were collected, including airspeed (anemometer; MS6252A Digital Anemometer System, Proster), air particle number (PM_2.5_ and PM_10_ combined; air quality meter; LKC-1000S+ Air Quality Monitor, Temtop), air gases (total volatile organic carbons and formaldehyde), and hutch noise level (decimeter phone app; Decibel X, SkyPaw Co.). Measures were taken from the side hutch door and targeted the center of the hutch approximately 0.5 m above the ground. Care was taken to minimally disturb the INT hutch environment while measures were taken.

Calf position and location was assessed using day- and night-vision trail cameras (SpyPoint ForcePro Trail Camera) that took pictures every 5 min and captured 3 calves per frame. Calf position and location, including calf lying outside (**OL**), standing outside (**OS**), or being inside (**IN**), were identified using computer vision systems described by Negreiro et al. (2022). The darkness inside the hutch prevented algorithm differentiation between lying versus standing inside the hutch. The objective of monitoring the aforementioned behavior was to determine if calves used the ventilation system and remained inside their hutch, particularly in warmer hours of the day. The period of time when calves were restricted EXT or INT was not included in the behavioral assessment.

All data were analyzed in SAS (v. 9.4, SAS Institute Inc., Cary, NC). The EXT environmental data were analyzed in PROC MEANS for descriptive statistics of daily average (average of all hours across one day) and hourly average (average of all days for each hour). Continuous INT environmental data, rectum surface temperature, and calf position and location were analyzed using PROC MIXED. Calf location and position was analyzed using PROC GLIMMIX with a binomial distribution and logit link function, where data were analyzed as a proportion of each event to the total number of events per hour. Results were expressed as the proportion of the hour either OS, OL, or IN. For all continuous data, the main effects included treatment, hour, period (1, 2, 3), and treatment × hour and period × treatment interactions. As a Latin square, the random effect was calf ID within period, treatment, and day. Hour was the repeated measure. The first-order autoregressive (AR-1) or compound symmetry (CS) covariance structures were used, and multiple comparisons were made by the Tukey test. The ACT treatment was in effect only when the ambient temperature was above 21°C, and therefore, data were only analyzed for hours of the day where T_db_ > 21°C. Some hours of the day (i.e., late night and early morning, 2100 to 0700 h) were consistently below this cutpoint. For the sake of visualization, data are reported across the full 24 h and reported in Figure 2, Figure 3, but only 0800 to 2000 h are considered results of active treatment. Hutch INT airspeed, air quality, noise level, and ΔINT-EXT RR, SR, RT, and ST were assessed at 1200 h and analyzed with PROC MIXED. Data analysis was similar to the above, except day was the main effect considered as the repeated measure instead of hour and the random effect was calf ID within period and treatment. An asterisk (*), cross (†), and ampersand (&) indicate a significant difference (*P* ≤ 0.05) between ACT and CON, ACT and PASS, PASS and CON treatments, respectively.

**Figure 2:**
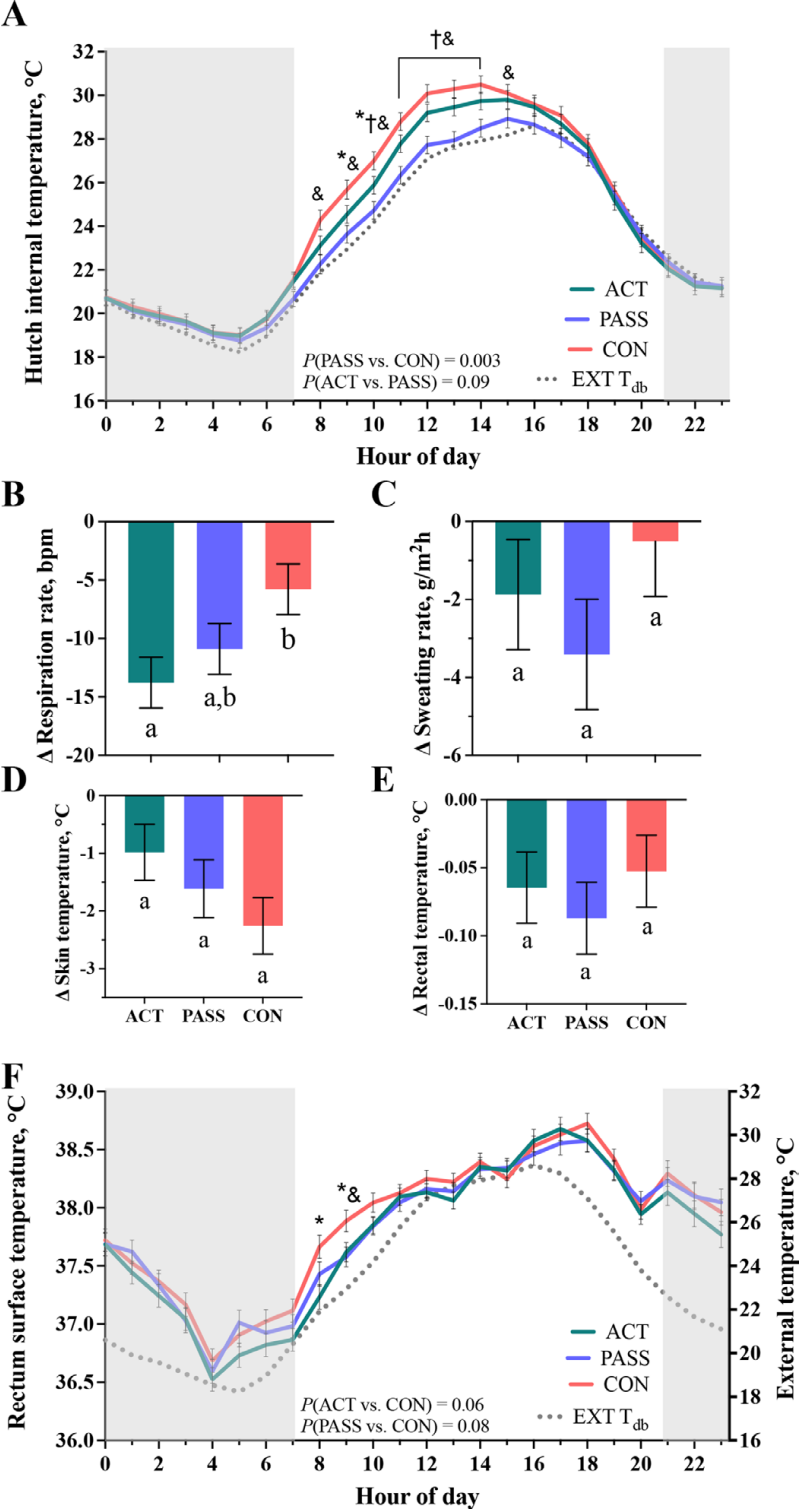
Effect of outdoor calf hutch ventilation treatments on hutch microclimate and calf thermoregulatory responses. (A) Hourly hutch internal (INT) temperature in each treatment group from 0800 to 2000 h compared with external (EXT) dry bulb temperature (T_db_). Calf respiration rate (B), sweating rate (C), skin temperature (D), and rectal temperature (E) assessed after a 30-min calf EXT restriction and INT restrictions at 1200 h (ΔINT-EXT) in hutches with no (CON), passive (PASS), or active (ACT) ventilation. (F) Hourly calf rectum surface temperature from 0800 to 2000 h. *, †, and & indicate a significant difference (*P* ≤ 0.05) between ACT and CON, ACT and PASS, and PASS and CON treatments, respectively. Disparate letters indicate a significant difference between treatment LSM. Error bars represent SEM.

**Figure 3:**
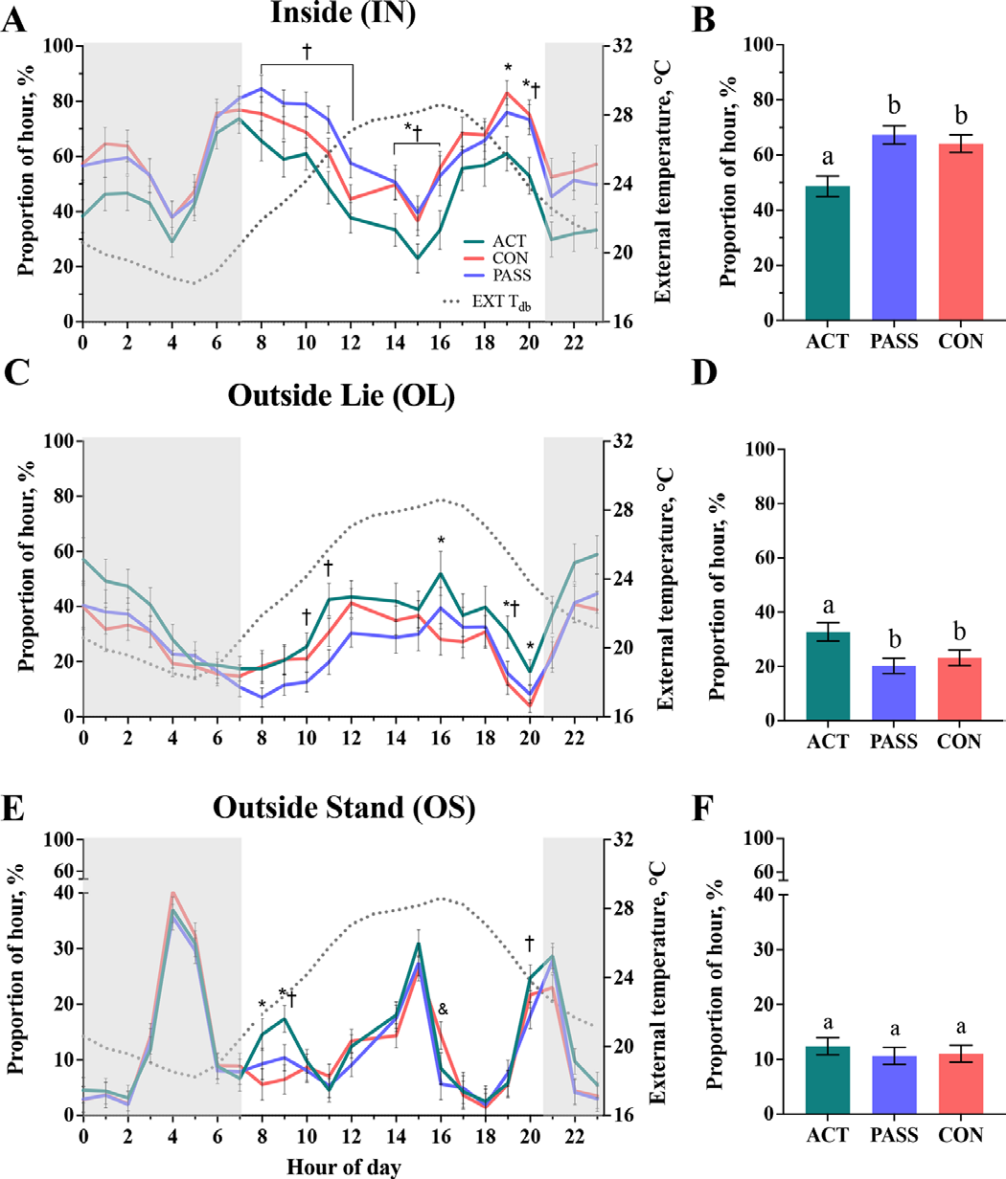
Effect of outdoor calf hutch ventilation on calf behavioral responses. The proportion of an hour that calves were inside (IN; A, B), outside lying (OL; C, D), or outside standing (OS, E, F) when calves were provided no (CON), passive (PASS), or active (ACT) ventilation. Data reported per hour and treatment LSM. *, †, and & indicate a significant difference (*P* ≤ 0.05) between ACT and CON, ACT and PASS, and PASS and CON treatments, respectively. Disparate letters indicate a significant difference between treatment LSM. Error bars represent SEM.

Daily average EXT T_db_ ranged from 20.1 to 26.8°C across the experiment with an average T_db_ of 23.3°C, RH = 73.1%, and THI = 71 across the experimental period (Figure 1D). Of the daily T_db_ averages, 84% of days remained above a T_db_ of 21°C, a potential threshold for thermal discomfort in continental dairy calves (Dado-Senn et al., 2023). When assessing hourly averages (Figure 2A), the daily minimum EXT T_db_ was between 0400 and 0500 h and averaged 17.5°C, while the daily maximum was between 1500 and 1600 h, which averaged 29.0°C across the experimental period.

Calf hourly hutch INT T_db_ were numerically greater than the EXT environment (average ACT: +1.1°C, PASS: +0.3°C, CON: +1.8°C; Figure 2A). The PASS ventilation had the lowest INT T_db_ (27.2 vs. 26.4 vs. 27.8°C for ACT, PASS, and CON), which was significantly lower than CON (*P* = 0.003) but only tended to be lower than ACT (*P* = 0.09). Specifically, PASS hutches decreased INT T_db_ relative to CON from 0800 to 1500 h and relative to ACT from 1000 to 1400 h (*P* < 0.05). The ACT hutches reduced INT T_db_ relative to CON at 0900 and 1000 h (*P* < 0.05; Figure 2A), though the treatment contrast was not different (*P* = 0.18). Hutch INT air speed was greater for ACT relative to PASS and CON (1.76 vs. 0.19 vs. 0.05 ± 0.09 m/s; *P* < 0.001), and PASS air speed was greater relative to CON (*P* = 0.04). The ACT led to elevated INT noise levels relative to PASS and CON (74.9 vs. 55.7 vs. 55.2 dB; *P* < 0.001). There were no treatment differences for number of air particles (*P* > 0.18) or air gases inside the hutch (data not shown, *P* > 0.65).

The change in calf physiological responses after EXT then INT restrictions at 1200 h are reported in Figure 2. Calves in ACT hutches had a significant reduction in RR relative to CON (−13.8 vs. −5.8 bpm; *P* = 0.01), but there was no difference between ACT and PASS calves (*P* = 0.35, Figure 2B). There were no treatment differences for changes in SR, ST, or RT (*P* > 0.19). Calves in CON hutches tended to have elevated rectum surface temperatures (38.24, 38.13, and 38.14°C for ACT, PASS, and CON, respectively; *P ≤* 0.08). At 0800 and 0900 h, CON calves had higher rectum surface temperature relative to ACT (*P* ≤ 0.03), but PASS and CON were only different at 0900 h (*P* = 0.01; Figure 2F).

Behavioral assessment found that calves housed in ACT hutches spent a greater proportion of each hour lying outside and a lesser proportion of each hour inside their hutches relative to PASS and CON calves (*P* ≤ 0.03; Figure 3A–F).

As expected, active ventilation via fans achieved elevated air speeds inside the hutch (1.76 m/s) relative to PASS (0.19 m/s) or CON (0.05 m/s) hutches. In mature dairy cattle, fan speeds around 1.7 m/s (calibrated to achieve a minimum of 1 m/s in each stall) improved cow thermoregulatory and productive responses within diminishing returns at 2.4 m/s, except for lying time (Reuscher et al., 2021a). However, there is a lack of research evaluating active ventilation at varying air speed levels to determine optimal outcomes in calves, particularly in a continental climate. Studies of alternative housing strategies and climates (i.e., indoor and subtropical) have investigated active ventilation via fans for heat abatement, reaching calf-level air speeds of 1.2 or 2.0 m/s with varying success in influencing animal-based and environmental outcomes (Dado-Senn et al., 2020; Montevecchio et al., 2022). The passive ventilation air speed reported in the present study is comparable to other studies promoting passive hutch ventilation (Moore et al., 2012; Reuscher et al., 2021b) at around 0.15 to 0.25 m/s.

Despite the substantial elevation of airspeeds, active ventilation did not improve hutch air quality relative to the other ventilation treatments, as determined by air particle number and select air gases. This is likely attributed to the function of outdoor hutch systems, which are known to have improved air quality relative to indoor housing (Nordlund, 2008). In a continental, indoor housing study, Hill et al. (2011) found no benefit to fan provision in reducing airborne bacteria concentrations, but in a subtropical climate, indoor pens provided active ventilation via fans had reduced ammonia concentration and total bacterial count relative to naturally ventilated pens (Montevecchio et al., 2022). These air quality parameters were not assessed herein and warrant additional investigation in this model.

Active ventilation reduced hutch INT T_db_ relative to CON, but only at 0900 to 1000 h. Meanwhile, PASS hutches had the lowest INT T_db_ relative to ACT and CON hutches, especially from 1000 to 1400 h as daily ambient temperatures began to rise. Thus, passive ventilation through upper and lower rear window openings best promoted a cooler hutch microclimate in the present study. Likely, the hutch design of ACT and CON (i.e., upper window closed) reduced the opportunity for rising warm air to escape, as achieved in the PASS hutches.

Notably, all ventilation strategies herein had greater hutch INT T_db_ relative to EXT ambient temperature, ranging from +0.3°C to +1.8°C. It is generally accepted that housing calves in a hutch system alters the microenvironment (Lago et al., 2006; Louie et al., 2018), with hutch INT T_db_ rising between 1.2°C to 13.5°C greater than the EXT environment, depending on hutch orientation, time of day, and solar radiation (Bakony et al., 2021). When assessing heat stress and health risks in hutch-housed dairy calves, it is important to account for microclimate, particularly if calves are not provided with ventilation. Instead of measuring the EXT environment to address microclimate variations, triggering active ventilation using a thermostat sensitive to hutch INT temperatures could be an avenue for future studies.

After hutch EXT and INT 30-min restrictions, calves in ACT hutches had a greater decline in RR relative to CON but not PASS calves, but there were no other differences in thermoregulation. Calves provided ACT also had reduced rectum surface temperature in the morning (i.e., 0800 and 0900 h) compared with calves in CON hutches, although not different from calves provided PASS. Together, these data suggest that active ventilation improved calf thermoregulatory responses compared with minimal ventilation but not passive ventilation. These results are similar to studies of continental, indoor (Hill et al., 2011) and arid, outdoor (Stott et al., 1976) individually housed calves in wire-panel hutches, where forced air movement improved both calf RR but not core body temperature (RT not reported in Hill et al., 2011). In a subtropical climate, indoor group-housed calves provided basket fans at the calf-resting level had reduced RR, RT, and ST and improved feed intake relative to calves provided natural ventilation (Dado-Senn et al., 2020). Conversely, individually housed calves in a similar environment did not improve thermoregulatory responses when provided active, ceiling fan ventilation versus natural ventilation (Montevecchio et al., 2022).

The effectiveness of active ventilation in altering calf thermoregulation depends on the type of ventilation provided, climate, housing strategy, and severity of heat stress. It is possible that the fluctuations in both diurnal and seasonal temperature in the present study reduced the risk for chronic heat stress exposure in calves, limiting the necessity for active over passive ventilation for heat abatement. However, the consistent air movement of active ventilation could be more reliable than passive ventilation, which relies on weather patterns and hutch orientation. Other factors contributing to the lack of active ventilation efficacy may include restriction assessment and fan timing. Modeling the effect of calf hutches on animal-based responses can be challenging, as calves are generally allowed free choice between inside and outside the hutch. The 30-min restriction might not be enough time for calves to respond to their microenvironments and thermoregulate accordingly. In addition, the thermostatically controlled fans may limit active ventilation effectiveness, whereas providing continuous ventilation independent of ambient temperature might promote more effective thermoregulation.

Calves under ACT ventilation spent a lesser proportion of each hour inside their hutch and a greater proportion lying outside their hutch relative to calves under PASS or CON, particularly during the cooler hours of the day. The reduction in hutch usage in ACT calves could partially explain the marginal impact of ACT on calf responses. Homeothermic mammals, including dairy calves, display thermoregulatory behaviors such as cool-seeking (i.e., lying near forced air movement or shade) as an initial response to rising ambient temperatures (i.e., thermal discomfort; Bianca, 1968; Van Os, 2019). Because calves did not voluntarily seek out active ventilation, calves could have been in an insufficient state of thermal discomfort to pursue heat abatement, especially if they perceived a tradeoff with disturbance in the hutch. It is possible that the fan provision was unsettling to calves due to excessive direct air movement and noise level inside the hutch. Alternatively, the airflow coming off the hutch (i.e., close to the hutch front door) was preferred. While there are no set thresholds for noise levels negatively affecting dairy calves, excessive noise is a source of abiotic environmental stress for dairy cattle (Mandel et al., 2016), leading to noise avoidance and altered activity levels. Future studies should investigate fans lessening noise levels and air speeds.

The present study's use of solar energy to power fans offers a potentially sustainable solution to rising global temperatures (Wang et al., 2020b). Photovoltaic technology (i.e., solar power) for dairy operations has been investigated for electric equipment demands, including fans for active ventilation (Gorjian et al., 2020; Wang et al., 2020b). Implementing a solar-powered fan system for dairy calves is currently not justifiable to many US dairy operations but may be an option for regions without reliable access to conventional electrical grids. In summary, providing passive or active ventilation to individual polyethylene calf hutches exerted positive effects over minimal ventilation on calf thermoregulation. Active ventilation via solar-powered fans improved hutch microclimate relative to minimal ventilation. Calves exposed to active ventilation had more robust thermoregulatory responses in specific time windows only when compared with calves provided minimal ventilation. These data suggest active ventilation is not advantageous over passive ventilation in cooling outdoor hutch-housed dairy calves. Additional investigation into fan design and its effect on calf productive outcomes is warranted.
